# Transmission potential of modified measles during an outbreak, Japan, March‒May 2018

**DOI:** 10.2807/1560-7917.ES.2018.23.24.1800239

**Published:** 2018-06-14

**Authors:** Kenji Mizumoto, Tetsuro Kobayashi, Gerardo Chowell

**Affiliations:** 1 Division of Epidemiology and Biostatistics, School of Public Health, Georgia State University, Atlanta, USA; 2 Graduate School of Medicine, Hokkaido University, Sapporo, Japan; 3 Division of International Epidemiology and Population Studies, Fogarty International Center, National Institutes of Health, Bethesda, USA

**Keywords:** epidemics, outbreak, measles, Japan, modified measles, modelling, transmission potential

## Abstract

A recent outbreak of measles in Okinawa Prefecture, Japan ended with 33 measles cases whose symptoms were masked because of insufficient protection against the disease (modified measles). Using quantitative modelling, we determined the transmission potential of measles by clinical presentation (classic vs modified measles). We found low ascertainment probabilities among modified measles cases, indicating that intensified public health interventions that specifically target this group should be implemented to better contain outbreaks with modified measles cases.

Japan has made great strides improving the vaccination coverage against childhood infectious diseases and, in 2015, the World Health Organization (WHO) verified that the country had eliminated measles [1,2]. However, in subsequent years several outbreaks originating from imported cases have been sporadically reported in Japan [3-5]. An outbreak of measles was reported in Okinawa Prefecture during March to May 2018, with the first (index) case reported on 20 March. The index case was a foreign traveller who visited Okinawa and had onset of symptoms on 14 March during his stay. The outbreak was declared over on 11 June 2018 [6]. A number of cases presenting with modified measles as a result of insufficient protection against the disease were reported during the outbreak [7]. As of 25 May 2018, there were a total of 99 autochthonous reported cases, primarily in Okinawa Prefecture. To investigate the extent to which human-to-human transmission is sustaining this outbreak, we quantified real-time changes in the transmission potential of measles in Japan using dynamic and statistical modelling tools.

## Epidemiological data analysis

We analysed laboratory-confirmed cases of the measles outbreak. For each case we collected information on age, residence, date of symptoms onset, vaccination history and symptomatic status (classic measles vs modified measles). Symptoms in modified measles cases are masked, so they do not present with the full (typical) symptoms of measles (fever, maculopapular rash and catarrhal symptoms such as cough, coryza or conjunctivitis) and their transmission risk is reported to be lower [8,9]. This difference in clinical presentation is due to suboptimal protection against the disease, which arises from an insufficient number of vaccination doses or the gradual loss of previously acquired immunity [9-12]. As a result of symptoms being masked, it can be harder to clinically diagnose cases; thus, modified measles cases are more often confirmed in a laboratory.

**Figure 1:**
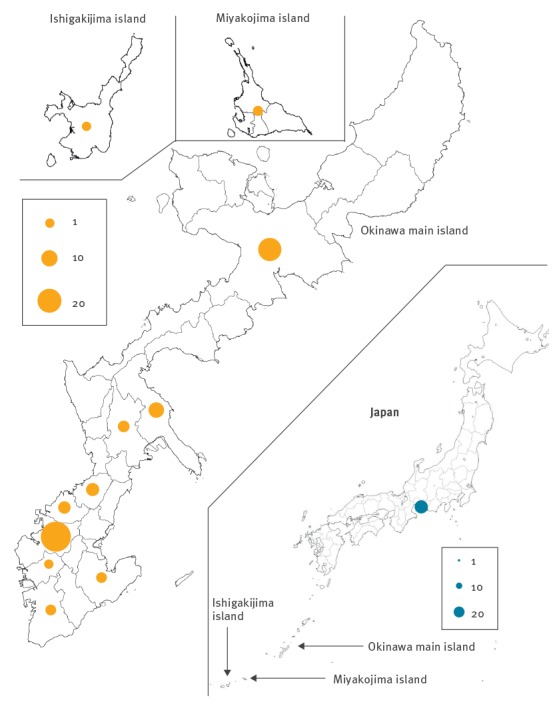
Geographical distribution of measles cases across districts of Okinawa Prefecture main island, Ishigaki island and Miyakojima island, Japan, 14 March–10 May, 2018 (n = 99)

Of the 99 cases notified as of 25 May 2018 [7], 40 were female and the age of cases ranged from under 1 year to over 50 years of age. Most cases (n=23) were reported from Naha city, the capital of Okinawa, located southwest of Japan’s main island (Figure 1). In addition to these cases, a further outbreak of 25 cases occurred in Aichi Prefecture, located on the main island of Japan; the primary case in this outbreak was an individual who had travelled to Okinawa Prefecture during the outbreak there.

Seventeen cases are not shown because the health centres did not report the specific locations. There were three cases in the north, seven in the middle and seven in the south of Okinawa Prefecture. Bottom-right shows the view of the whole of Japan at a larger scale, indicating cases imported from Okinawa.

**Figure 2:**
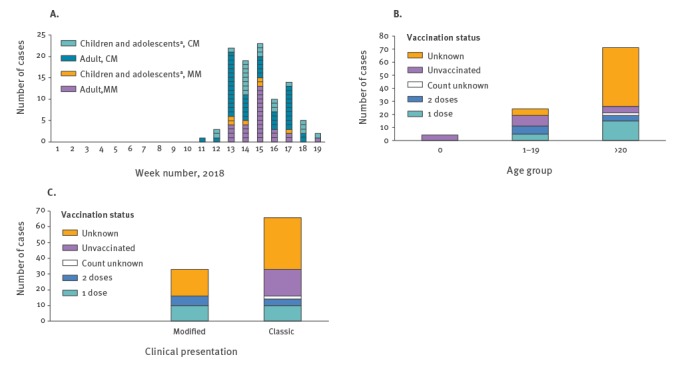
Number of measles cases (A) by week, age and clinical presentation, (B) by age group and vaccination status, (C) by clinical presentation and vaccination status, Okinawa Prefecture, Japan, 14 March–10 May 2018 (n=99)

CM: Classic measles; MM: Modified measles.

^a ^Aged < 20.

The weekly number of cases in Okinawa can be seen in Figure 2A. Measles cases peaked in week 15. There were 33 modified cases overall, with a higher number of modified cases in adults (n=27) than in children and adolescents (here defined as under 20 years of age) (n=6). The age distributions of cases stratified by vaccination history are presented in Figure 2B. Of the 99 cases reported in Okinawa, four were infants under one year of age, 24 were aged 1–19 years and 71 were 20 years and older. In total, 32 cases (32%) had been vaccinated against measles: 20 with one dose, 10 with two doses and two with verified vaccination but the number of doses unknown. Seventeen cases had not been vaccinated, while 50 did not know their vaccination status. Of these 99 cases, 33 (33%) presented with modified measles and their vaccination status can be seen in Figure 2C.

## Epidemiological modelling

We conducted real-time analysis of the transmission dynamics of the outbreak using mathematical and statistical modelling. We employed a discrete-time age-structured integral equation model of the transmission dynamics through state-space modelling. We stratified the time series of confirmed cases into four subgroups by age (children and adolescents (age < 20) and adults (age ≥ 20)) and clinical presentation (classic and modified measles) to assess real-time transmission potential.

Let *f_s_* denote the probability mass function of the serial interval of measles, e.g. the time from illness onset in a primary case to illness onset in the secondary case, of length *s* days, given by:

$$f_{s}=G\left(s\right)-G\left(s-1\right)\;.$$

For s > 0, G(.) represents the cumulative distribution function of the gamma distribution. We characterised the expected number of new infections E[*c_i_,_a_*(t)] in the age group *a* of measles type *i* at onset day *t* as follows,

$$E\left[c_{i,a}\left(t\right)\right]=\sum_{s=1}^{\infty }\sum_{i=1}^{j}\sum_{b=1}^{k}{E[c}_{b}(t-s)]f_{s}{\delta R}_{i,ab}\left(t\right),$$

where *R_i_,_ab_* is the product of *R_i_*, and *m_ab_*, where *R_i_* represents the average number of secondary cases generated by measles type *i* (classic or modified) and *m_ab_* is the contact frequency of individuals in age group *a* with those in age group *b* (assumed to be known [13]); let *M* be square matrices, the (*a*, *b*) element of *M* is *m_a,b_* and assumes a frequency-dependent contact rate. More detailed contact heterogeneities, e.g. the type and duration of contacts, are not considered in our model. *k* ( = 2) is an index for the number of age groups. We model the time-dependent variation in the *R_ab_* to incorporate the impact of public awareness about the outbreak and the implementation of extensive contact-tracing efforts using a time-dependent parameter:

$$\delta =\left\{\begin{array}{ccc} 1 & for & t<t_{0}\\ \alpha_{1} & for & t_{0}\leq t<t_{1}\\ \alpha_{2} & for & t_{1}<t \end{array}\right.$$

where *t*_0_ and *t*_1_ represent the dates when secondary transmission within Okinawa Prefecture was detected on 29 March 2018 and the official public health alert level was raised on 3 April 2018.

For classic and modified measles cases, we used zero-inflated Poisson modelling to model an age-dependent and type-dependent probability of occurrence, *q_i,a_* [14], assuming that the number of observed cases of type *i* in age group *a* at day *t*, denoted by *h_i_,_a_*(*t*), is the result of a Bernoulli sampling process. The expected value is denoted by E(*c_i,a_*;*H*_t-1_); the conditional expected infection in type *i* among age group *a*, on day *t*, given the observed time series data from day 0 to day (*t*-1) is given by *H_t_*_-1_. Thus, the expected number of newly observed cases is written as follows:

$$E\left[h_{i,a}\left(t\right);H_{t-1}\right]=\{\begin{array}{ccc} \left(1-q_{i,a}\right)+q_{i,a}E\left[c_{i,a}\left(t\right);H_{t-1}\right], & & if\;h_{i,a}=0\;,\\ q_{i,a}E\left[c_{i,a}\left(t\right);H_{t-1}\right], & & otherwise, \end{array}$$

We also take into account the ascertainment bias (not all infected seek care) and we assume that the number of reported cases of type *i* among subgroup *a* on day *t*, *h_i,a_*(t), is the product of the age-dependent and type-dependent ascertainment rate, *θ_i,a_*, and the actual laboratory-confirmed number of cases, *c_i,a_*(t), (not all the cases are diagnosed/reported). The expected number of newly observed cases are given by:

$$E\left[h_{i,a}\left(t\right);H_{t-1}\right]=\{\begin{array}{ccc} \left(1-q_{i,a}\right)+q_{i,a}\theta_{i,a}E\left[c_{i,a}\left(t\right);H_{t-1}\right], & & if\;h_{a}=0\;,\\ q_{i,a}\theta_{i,a}E\left[c_{i,a}\left(t\right);H_{t-1}\right], & & otherwise, \end{array}$$

Here we assume that the incidence function, *h_i,a_*(*t*), follows a Poisson sampling process with expected value E[*h_i,a_*]. The likelihood function for the time series of observed cases that we employ to estimate the effective reproduction number *R*_t_ and other relevant parameters is given by:

$$L\left(U;H\right)=\prod_{t=1}^{T}\prod_{i=1}^{j}\prod_{a=1}^{m}\frac{E{\left(h_{i,a}\left(t\right);H_{t-1}\right)}^{h_{i,a}\left(t\right)}\exp\left(-E\left(h_{i,a}\left(t\right);H_{t-1}\right)\right)}{h_{i,a}\left(t\right)!}.$$

where *U* indicates parameter sets that are estimated from the likelihood.

We derive the instantaneous time-dependent effective *R_t_* for classic and modified measles from the largest eigenvalue of the age-dependent next generation matrix (NGM). The serial interval is characterised using a gamma distribution with the mean and SD at 11.8 and 2.0 days, respectively [15]. We fixed the maximum value of the serial interval at 16 days as the cumulative probability distribution of the gamma distribution at 16 days is 0.985.

We estimated model parameters using a Monte Carlo Markov Chain (MCMC) method in a Bayesian framework. Point estimates and the corresponding 95% credibility intervals (CrI) are drawn from the posterior probability distribution of each parameter.

## Findings from the real-time outbreak analysis

**Figure 3:**
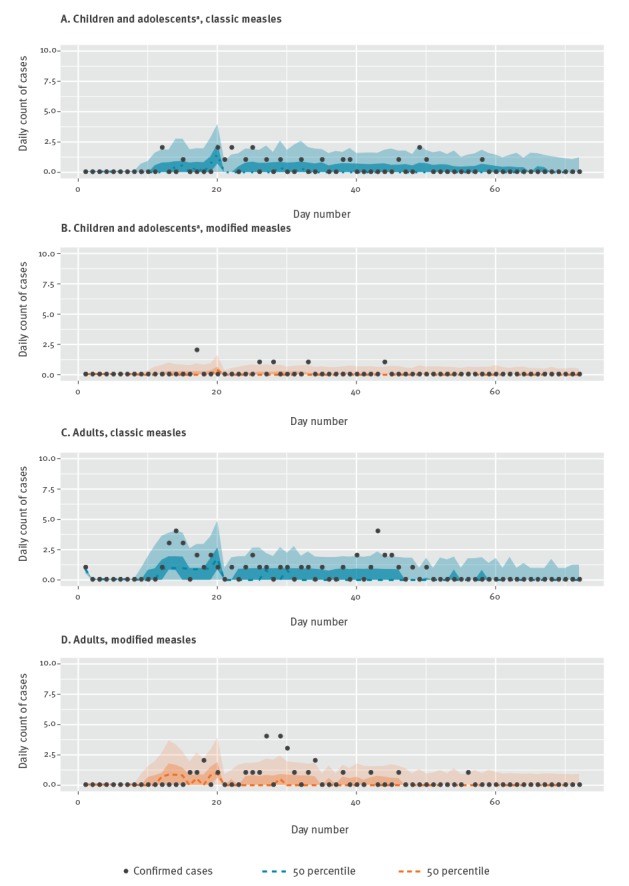
Observed and estimated number of measles by age, (A) children and adolescents^a^ with classic measles, (B) children and adolescents^a^ with modified measles, (C) adults with classic measles, (D) adults with modified measles, Japan, 14 March–10 May 2018 (n=99)

^a ^Aged < 20.

Observed and posterior estimates of the total daily number of classic and modified measles cases by age groups are shown in Figure 3. We estimated that the total burden of measles cases (cumulative incidence) by subgroups are 4.9 (50% CrI: 1.0‒40.0) (95% CrI: 0.0‒113.5) for classic measles cases among children and adolescents, 0.8 (50% CrI: 0.2‒7.9) (95% CrI: 0.0‒45.0) for modified measles cases among children and adolescents, 12.3 (50% CrI: 3.6‒42.6) (95% CrI: 0.6‒131.4) for classic measles cases among adults and 6.6 (50% CrI: 0.9‒30.1) (95% CrI: 0.0‒102.5) for modified measles cases among adults. For comparison, the number of reported confirmed cases during our study period were 27, 6, 44 and 22, respectively.

Light orange/blue and dark orange/blue indicates 95% and 50% credible intervals for posterior estimates, respectively.

**Figure 4:**
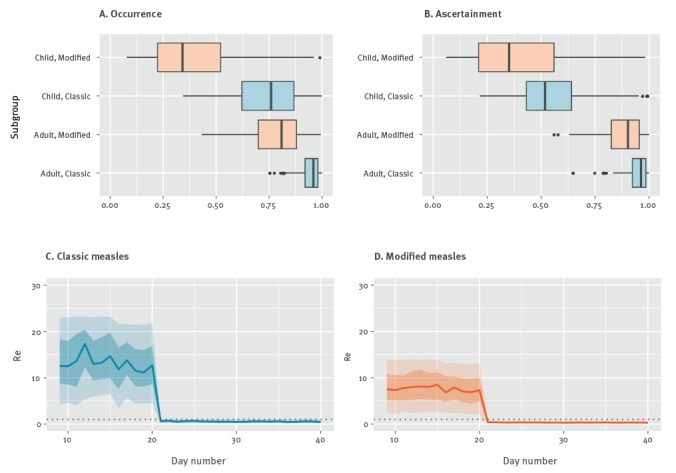
Probabilities of measles cases (A) occurrence, (B) ascertainment, and time–dependant effective reproduction number of (C) classic measles, (D) modified measles, Japan, 14 March–10 May 2018 (n=99)

^a^ Aged < 20.

Estimates for the probability of occurrence and ascertainment probabilities by subgroup are shown in Figure 4(A and B) Our real-time estimates of *R*_t_ with quantified uncertainty during the study period are displayed in Figure 4 (C and D). Overall, *R*_t_ started well above the epidemic threshold of 1.0, but it declined below the threshold in the midst of outbreak, probably as a result of control interventions (Figure 4 C and D). This is also supported by our sensitivity analyses where we examine how *R*_t_ is affected by small variations in the mean serial interval ranging from 9.8 to 13.8. At the early phase before the intervention, the ratio of *R*_t_ in classic measles to *R*_t_ in modified measles is estimated to be 1.74 (50% CrI: 1.15‒2.78) (95% CrI: 0.49‒6.39). Moreover, the time-dependent scaling factor quantifying the extent of effectiveness of interventions, *α*_1_ and *α*_2_, are estimated to be 0.92 (50% CrI: 0.87‒0.97) (95% CrI: 0.69‒1.00) and 0.04 (50% CrI: 0.04‒0.05) (95% CrI: 0.04‒0.05), respectively.

The average number of secondary transmission were calculated from the dominant eigenvalue of age-dependent next-generation matrix.

Horizontal grey dashed line indicates the reproduction number at 1.0 for reference, below which the epidemic follows a declining trend.

Day 1 is 14 March 2018, the first day of the outbreak.

## Discussion

We examined the effective reproduction number of classic and modified measles for the recently terminated outbreak in Okinawa Prefecture, Japan which had 33 modified measles cases. The outbreak was officially declared over on the 11 June 2018 [6]. Using current epidemiological data, we also estimated probabilities of occurrence and ascertainment probabilities for classic and modified measles cases. *R*_t_ has declined below the threshold of 1.0 after day 21 (3 April), indicating measles transmission is being contained as a result of enhanced contact tracing, health surveillance efforts and vaccination catch-up.

At the beginning of the outbreak, the effective reproduction number of modified measles was above the epidemic threshold, but lower compared with classic measles; modified measles cases also had lower probabilities of occurrence and ascertainment for both age groups. This suggests that modified measles play a role in the transmission dynamics of measles outbreaks. They are likely a result of insufficient vaccination doses or loss of natural boosting opportunities, especially among older children [16,17].

Several outbreaks have been reported in a number of European countries since 1 February 2017, as a result of suboptimal vaccination coverage [18,19]. Measles affects all age groups across Europe, but 50% (n=6,788) of cases (of known age) were over 15 years old as of April 2018 [19]. Of 12,111 cases, the vast majority, 87% were unvaccinated, 10% had received two or more doses and the remaining either did not know number of doses or their vaccination status [19].Vaccination campaigns that specifically target unvaccinated or partially vaccinated population groups could, therefore, help prevent and contain outbreaks such as the one affecting Okinawa Prefecture.

Our model does not capture the transmission dynamic of modified measles in adults at the early phase of outbreak, this could have resulted in some missed cases due to the presence of masked symptoms. It could also suggest that there are differences in the infection process between transmission from the primary case in an individual infected abroad to the local community and then subsequently autochthonous/local transmission in the community. The impact of modified measles on transmission dynamics might be underestimated due to the possible delay in available information on modified measles. To account for this, we assumed that the serial interval and contact frequency is invariant across age groups, regardless of vaccination history.

It has been shown previously, that vaccinated cases, presenting with classic measles, appear to be less contagious than unvaccinated cases [10].The proportion of vaccinated cases among the total cases in our study, including those with unknown vaccination history, indicate that the vaccinated population may play a role in the transmission dynamics of measles – probably due to secondary vaccination failure (waning of vaccine-induced immunity to non-protective levels) [20] or owing to enhanced transmission at the household level (e.g. continual intense exposure).
